# ‘Never testing for HIV’ among Men who have Sex with Men in Viet Nam: results from an internet-based cross-sectional survey

**DOI:** 10.1186/1471-2458-13-1236

**Published:** 2013-12-28

**Authors:** Macarena Cecilia García, Quyen Le Duong, Licelot Eralte Mercer, Samantha Beth Meyer, Paul Russell Ward

**Affiliations:** 1 Doctoral Candidate, Discipline of Public Health, Flinders University, Adelaide, Australia; 2 PEPFAR Program Director, U.S. Agency for International Development, Maseru, Lesotho; 3 Independent Consultant, Hanoi, Viet Nam; 4 Allan Rosenfield Global Epidemiology Fellow, Association of Schools of Public Health, Maseru, Lesotho; 5 Discipline of Public Health, Flinders University, Adelaide, Australia; 6 38 Sunset Ridge Circle, Pomona, CA 91766, USA

**Keywords:** Men who have Sex with Men, High risk HIV behaviours, HIV testing, Viet Nam

## Abstract

**Background:**

Men who have sex with men in Viet Nam have been under-studied as a high-risk group for HIV infection, and this population’s percentage and determinants of HIV testing have not been comprehensively investigated.

**Methods:**

A national Internet-based survey of self-reported sexual and health seeking behaviours was conducted between August and October 2011 with 2077 Vietnamese men who had sex with men in the last twelve months to identify the frequency of ‘never testing for HIV’ among Internet-using MSM living in Viet Nam, as well as the factors associated with this HIV-related high-rish behavior. Logistic regression analyses were conducted to assess the demographic characteristics and behaviours predicting never testing for HIV.

**Results:**

A total of 76.5% of men who have sex with men who were surveyed reported never having been voluntarily tested for HIV. Predictors of never being tested included having a monthly income less than VND 5 Million, being a student, using the Internet less than 15 hour per week, and not participating in a behavioural HIV intervention.

**Conclusions:**

Never testing for HIV is common among Internet-using men who have sex with men in Viet Nam. Given the dangerously high prevalence of this high-risk behaviour, our findings underscore the urgent need for segmented and targeted HIV prevention, care and treatment strategies, focusing on drastically reducing the number of men who have sex with men never testing for HIV in Viet Nam.

## Background

The HIV epidemic in Viet Nam is a concentrated one, with the highest prevalence found among key populations at higher risk of infection; these include injecting drug users (IDU), female sex workers (FSW), and men who have sex with men (MSM) [1]. As documented by the Ministry of Health (MOH) 2009 Estimates and Projections Report, [2] MSM populations are larger than those of other key populations in Viet Nam, and are primarily concentrated in urban areas such as the Mekong River Delta (73,727), Ho Chi Minh City (64,247), the Red River Delta (60,698), and Ha Noi (35,436) [2]. In these areas, IDU and FSW population estimates are lower than size estimations of MSM.

According to recent results of the 2011 Viet Nam HIV/AIDS Estimates and Projections Report, adult HIV prevalence was 0.45% in 2011. It is estimated there will be up to 263,317 People Living with HIV (PLHIV) by 2015 [3]. Unlike Thailand to the west, the epidemic in Viet Nam is not as severe. UNAIDS [1] reports that the epidemics in Ho Chi Minh City and in the northeast coast began earlier, while epidemics in other parts of the country are much more recent. Variability in geographic distribution and concentration of HIV cases show a concentrated epidemic in large cities and densely populated provinces. In urban centers, the HIV epidemic is disproportionately concentrated in IDU, FSW and MSM populations [1].

Male-to-male sexual contact has been an important route of HIV-1 infection since HIV was first identified. In the past few years, there has been increasing concern about new, newly identified, and resurging epidemics of HIV infection in MSM on a global level [4,5]. Against the backdrop of low and declining adult HIV prevalence in most countries, MSM continue to be disproportionately affected by HIV infection [4]. In Asia, a man who has sex with another man is 18.7 times more likely to become infected with HIV than someone from the general population [4]. In recent years, epidemiological research has identified high HIV prevalence among MSM in several Asian countries [6]. Recent data made available through the Vietnam Integrated Biological and Behavioral Survey (IBBS) suggest a significant increase in HIV prevalence among MSM in both Ha Noi and Ho Chi Minh City. For MSM who had sold sex to men in Ha Noi, HIV prevalence rose to 14% in 2009 from 9% in 2006. For MSM who had not sold sex, prevalence was 20% in 2009, versus 11% in 2006. The data for Ho Chi Minh City were similar [7].

Rapidly rising HIV prevalence among MSM in Viet Nam draws concern from the region and around the world. According to a recent World Bank report [8], there are key behaviours that put MSM at greater risk of HIV infection, such as high numbers of multiple and concurrent sexual partners, unprotected anal intercourse, and low percentages of HIV testing. In a recently published systematic review, García, Meyer and Ward assert, “although HIV counselling and testing is increasing among IDU and FSW [in Viet Nam], it remains inadequately low among MSM, and has not increased since the 2006 round of IBBS” [9]. Beyrer, Wirtz, Walker, et al. strongly recommended voluntary counselling and testing (VCT) as a key strategy for inclusion in comprehensive HIV prevention interventions for MSM in their 2011 World Bank report, citing that “VCT provides an important engagement point between MSM and health care providers…” [8]. This paper seeks to identify the frequencies and key determinants of never testing for HIV, and therefore aims to fill the knowledge gap that exists regarding the prevalence and predictors of this HIV-related high-risk behaviour among MSM.

## Methods

### Study design and site selection

Internet access is dramatically increasing in Viet Nam. According to Nguyen et al., [10] as of June 2009, 21.5 million Vietnamese were using the Internet, approximately one-fourth of the total population. Although Internet users are concentrated in urban areas, one study found that 30% of rural MSM have sought a sexual partner via the Internet [10]. Face-to-face interviews were not conducted in this study, as this data collection method would have potentially limited the number of MSM reached, as well as restricted the candidness of responses. As Liamputtong asserts, “online research provides possibilities to reach a terrain of vulnerable participants, such as…people from socially marginalized groups such as gays and lesbians, who may not be easily accessed in face-to-face research methods” [11].

A computer-assisted survey using a self-administered questionnaire was deemed by the researchers as the most appropriate and effective tool to obtain candid responses from a highly stigmatized population. The method allowed participants to respond openly without the possibility of being influenced by an external interviewer. This was particularly important due to the sensitive sexual and behavioral nature of the survey questions.

### Study population

Individuals eligible to participate in this study were Vietnamese men 18 years or older who reported having had sex (hand and/or oral and/or penetrative) with a man in the last 12 months, and who were living in Viet Nam at the time of the study. The specific inclusion and exclusion criteria allowed the researchers to better meet the study’s aims and objectives by including responses from MSM who did not consider themselves homosexual. This is an important distinction in Viet Nam because a large proportion of MSM do not consider themselves homosexual, and therefore may be at greater risk of HIV infection [1].

### Participant recruitment

Formal permission was sought, and rights to commercial space for banner advertisement with a customized imbedded Internet link to the survey instrument was granted by the administrators of the 12 most popular and highly visited MSM commercial and social networking websites in Viet Nam. Banner advertisement included a graphic of an unidentifiable Asian man, with primary tagline reading: ‘30 minutes for looking back at yourself, a study of sexual behaviours, multiple concurrent sexual partnerships and HIV risk factors’. The researchers provided an incentive for participation consisting of a contribution – of US $0.50 – to a Vietnamese civil society organization active in gay and lesbian advocacy per each completed questionnaire. The instrument remained active and accessible online for a period of two months (August 10 to October 10, 2011) to ensure sample size achievement. Those who clicked the recruitment banner were routed to a secure study website. Potential participants were instructed to read the informed consent describing the aim of the study and to click on an ‘agree’ button to confirm consent. Participants were then provided access to the survey, which required 20–25 minutes to complete.

### Sample size

This study used a nonprobability method, which consisted of purposive sampling for participant accrual. Given the cultural sensitivity around homosexuality within Vietnamese society, [12] the decision was made to use purposive sampling utilizing a confidential Internet-based self-administered questionnaire. The sampling method applied did not require public MSM self-identification, which may have placed participants at risk; rather, method protected the anonymity and privacy of study participants. Purposive sampling is appropriate for vulnerable populations and lends itself to the targeting of participants in a discrete manner, being referred to as one of several “non-invasive sampling strategies” [13].

#### Data collection

### Study instrument

A comprehensive quantitative instrument containing approximately 110 questions was developed in consultation with a variety of key experts and practitioners active in MSM research in Viet Nam. The survey tool was designed to include questions resulting in data triangulation and validation. The instrument was designed by the researchers in the English language, translated into Vietnamese by a professional translator working in the HIV/AIDS field in Viet Nam, and then translated back to English. A second Vietnamese HIV specialist verified the English – Vietnamese – English translation. The questionnaire was in the Vietnamese language. Domains of this instrument included demographics, weekly Internet-usage, relationship patterns, sexual behavior, self-reported HIV sero-status, health-seeking practices, and existing HIV knowledge and risk perception. The questionnaire was part of a comprehensive study on MSM behaviors and HIV risk in Viet Nam. This paper focuses only on frequencies and associated factors of never testing for HIV among MSM in Viet Nam.

### Statistical methods for data analysis

The data set was analyzed with IBM® SPSS® 19.0 statistical analysis software version 20.0.0 (IBM Corporation, Armonk, NY) with the significance level of statistical tests fixed at 0.05. Descriptive data is presented with various denominators as a result of different non-response rates in the survey and data omitted by researchers due to inconsistent responses validated during data cleaning process.

High level of non-response was observed across all questions presented in the survey, and thus missing data is substantial across variables. To define socio-behavioral and demographic factors associated with ‘Never testing’ for HIV univariate logistic regression models were conducted to determine which were significantly associated. Independent variables of the models included well-established demographic characteristics and sexual behaviors (i.e. number of sex partners, unprotected anal intercourse, consuming alcohol before and/or during sex) identified from peer-reviewed literature. Multicollinearity was assessed to ensure that statistically significant factors identified from univariate models were not highly correlated among one another (coefficient −0.8 ≤ *Rho* ≥ 0.8). Variables found to be statistically associated at *p-value* of ≤ 0.05 were included in the multivariable model, which formed the core model tested. The model was tested by systematically plugging in variables in step-wise fashion. The final adjusted model was selected after identifying confounders, which were identified by a change in the Regression Coefficient in the core model of 10% or greater. Finally, adjusted Odd Ratios (aOR) were calculated for the independent variables of interest. Using a multivariable logistic regression core model, the association of the dependent variable (‘Never testing’ for HIV) was tested with significant independent variables and confounders previously identified.

### Ethical consideration and funding

Ethical approval was sought and granted from the Ha Noi School of Public Health Institutional Review Board, a Viet Nam governmental entity, and the Flinders University Social and Behavioural Research Ethics Committee. This study was funded by two Flinders University post-graduate research grants.

## Results

### Enrolment

A total of 5128 individuals responded to the survey. After inclusion and exclusion criteria were applied and data checks were conducted, the number of eligible participants was reduced to 2077 (see Table 1).

**Table 1:** inclusion criteria (N = 5,128)

**Inclusion criteria**	**% (n)**
Vietnamese citizen residing in Viet Nam	88.9 (4561)
Male (Identity)	88.7 (4546)
Male (Biological)	84.1 (4314)
Past Research Participation	81.9 (4202)
18 Years or Older	78.2 (4008)
Intercourse with Men (≤ 12 mos)	56.0 (2872)
**All Inclusion Criteria Met**	**40.5 (2077)**

### Population demographics

The majority of eligible participants completing the questionnaire came from three websites, Tao Xanh (17.0%), Tinh Yeu Trai Viet (15.7%), and Vietboy.net (14.6%). The participants who were surveyed were from 54 of the 63 provinces located in Viet Nam; however, 77.4% hailed from five of the larger, more populated regions in Viet Nam including Ho Chi Minh City (60.8%), Ha Noi (10.2%), Can Tho (3.4%), Da Nang (1.9%), and Hai Phong (1.1%). Demographic characteristics are presented in Table 2.

**Table 2:** Demographics

**Demographics**		**Total% (n/N)**
Sex	Male	96.1 (1996/2077)
	Male-to-female (sex change)	0 (1/2077)
	Intersex or transgender	3.9 (80/2077)
Age^†^	18–20	30.3 (628/2074)
(years)	21–25	41.1 (852/2074)
	26–30	15.5 (322/2074)
	31–35	7.1 (147/2074)
	36–40	3.2 (66/2074)
	Older than 40	2.8 (59/2074)
Marital Status	Single	92.4 (1878/2033)
	Married	6.2 (127/2033)
	Separated or Divorced or Widow	1.4 (28/2033)
Education	Did not go to school	0.4 (8/2061)
	Completed primary school only	0.1 (3/2061)
	Completed up to secondary school	2.5 (52/2061)
	Completed up to high school	25.2 (520/2061)
	Completed up to university, college or vocational school	63.5 (1309/2061)
	Completed post-graduate level	8.2 (169/2061)
Occupation	Student	44.0 (905/2056)
	Office workers or sales clerk	32.5 (668/2056)
	Self-employed	8.5 (175/2056)
	Government employee	6.9 (142/2056)
	Laborer	3.8 (79/2056)
	Sex worker	0.5 (10/2056)
	Other	3.7 (77/2056)
Salary (monthly)^††^	No income	20.8 (425/2044)
	Less than VND 1,000,000	9.5 (195/2044)
	VND 1,000,000–5,000,000	38.6 (789/2044)
	Above VND 5,000,000–10,000,000	18.6 (380/2044)
	More than VND 10,000,000	12.5 (255/2044)
Internet Usage (weekly)	Do not use Internet	0.3 (7/2057)
	Less than 3 hours	6.5 (134/2057)
	3–7 hours	14.9 (307/2057)
	8–12 hours	16.7 (343/2057)
	13–15 hours	13.0 (268/2057)
	16 hours or more	48.5 (998/2057)

^†^The survey included two separate questions about age: 1) screening question: are you 18 years or older? 2) main questionnaire: what is your age? Three cases responded ‘Yes’ to the screening question but did not respond to the age question in the main questionnaire.

^††^Average USD/VND exchange rate during data collection period: 20,992.54 (Source: http://Oanda.com).

### MSM archetypes

Most of the participants surveyed in this study self-identified as being homosexual (78.1%), 20.4% as being bisexual, 1.2% as being heterosexual, and only 0.2% as being transgender. Two-thirds of MSM surveyed described themselves as *bong kin*, or MSM who have a masculine appearance and wear men’s clothing and who usually keep their sexual preferences secret to avoid both stigma and discrimination [12]. A small percentage of respondents (2.6%) described themselves as *bong lo*, or MSM who have a feminine appearance, wear women’s clothing and are openly gay [12].

### HIV testing

**Figure 1:**
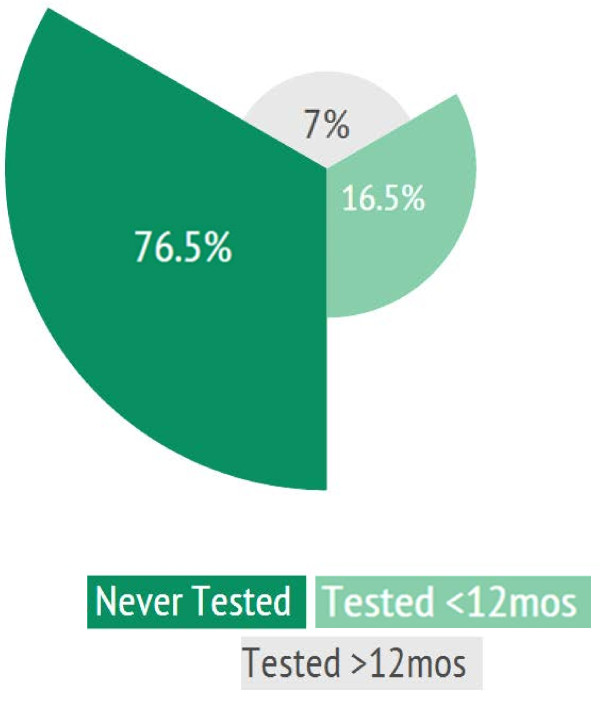
HIV Testing among MSM in Viet Nam (%).

A majority of MSM reported never having had a voluntary HIV test (76.5%; 1187/1552) (Figure 1).

**Figure 2:**
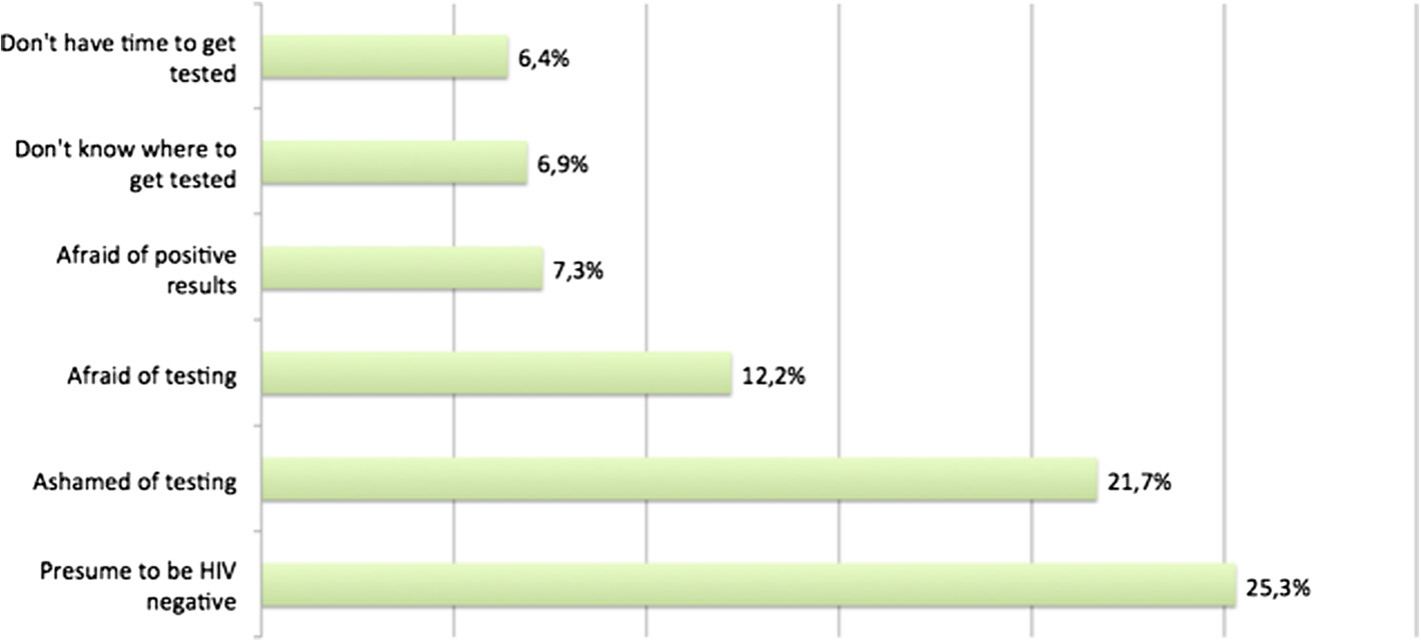
Reasons for ‘Never Testing’ for HIV, N = 641.

Participants indicated varied reasons for not being tested for HIV. The most common reasons are listed in Figure 2. Other reasons included not believing the test is necessary, fear of stigma and discrimination, and hesitation of disclosing information.

Of those who reported that they had a voluntary HIV test, 45.8% (521/1137) stated that they were HIV negative; 3.3% (37/1137) stated that they were HIV-infected; and 50.9% (579/1137) stated that they did not know the results of their HIV test. In this study 64.8% (820/1265) of MSM reported knowing where to get voluntarily tested for HIV.

Table 3 provides an overview of the factors associated with never testing for HIV.

**Table 3:** Risk factors associated with ‘never testing’ for hiv

	**Univariate logistic regression analysis**			**Multivariate logistic regression (final models for each independent variable)**		
**Factors**		**OR**			**aOR**	
	**Beta**	**(95% CI)**	**p-value**	**Beta**	**(95% CI)**	**p-value**
Age ≥ 25 years	−0.84	0.43*		−0.17	0.84	
		(0.34 - 0.55)	<0.001		(0.52–1.38)	0.50
Homosexual (self-reported)	0.22	1.25		0.41	1.51	
		(0.97 - 1.61)	0.09		(0.94–2.40)	0.09
Bong Kin (self-reported)	−0.14	0.87		0.09	1.09	
		(0.67 - 1.12)	0.28		(0.69–1.73)	0.72
Bisexual	−0.09	0.92		0.10	1.10	0.65
		(0.73 - 1.16)	0.48		(0.73–1.65)	
Education (university, vocational school or higher)	−0.66	0.52*		−0.33	0.72	
		(0.39 - 0.69)	<0.001		(0.42–1.23)	0.23
Marital Status (Married)	−0.44	0.64		0.31	1.36	
		(0.40 - 1.03)	0.07		(0.50–3.69)	0.54
Monthly income (≤ 5 million VND)	1.12	3.07*		0.69	1.99*	
		(2.41 - 3.93)	<0.001		(1.22–3.23)	0.006
Student	0.96	2.62*		0.63	1.88*	0.02
		(2.03 - 3.38)	<0.001		(1.09–3.24)	
Urban Resident	−0.51	0.60*		−0.23	0.79	
		(0.44 - 0.84)	0.002		(0.46–1.38)	0.41
Internet Use (≥ 15 hrs/wk)	−0.33	0.72*		−0.50	0.61*	
		(0.57 - 0.91)	0.007		(0.40–0.92)	0.02
Internet Solicitation for Casual Sex Partners	0.14	1.15		0.02	1.02	
		(0.88 - 1.49)	0.32		(0.63–1.65)	0.95
Anal Sex (≤ 6 mos)	−0.46	0.63*		−0.41	0.67	
		(0.48 - 0.84)	0.001		(0.39–1.14)	0.14
Alcohol Consumption (before and/or after sex)	−0.33	0.72*		0.05	1.05	
		(0.56 - 0.92)	0.01		(0.68–1.62)	0.84
Medically Circumcised	0.20	1.22		0.53	1.69	
		(0.83 - 1.80)	0.31		(0.91–3.14)	0.10
Accessed free condoms (≤ 12 mos)	−0.80	0.45*		−0.31	0.73	0.32
		(0.32 - 0.63)	<0.001		(0.40–1.35)	
Accessed free water-based lubricant (≤ 12 mos)^†^		0.50*			1.37	
	−0.70	(0.33 - 0.75)	0.001	0.31	(0.54–3.45)	0.51
Received HIV Education Materials (general ≤ 12 mos)	−0.41	0.66*		−0.43	0.65	0.11
		(0.50 - 0.87)	0.003		(0.39–1.10)	
Accessed HIV Education Materials (MSM brochures ≤ 12 mos)	−0.63	0.53*		0.29	1.33	
		(0.38 - 0.74)	<0.001		(0.66–2.66)	0.42
Contacted by MSM Peer Educator (≤ 12 mos)	−0.91	0.40*		−0.45	0.64	0.25
		(0.26 - 0.62)	<0.001		(0.30–1.36)	
Participation in HIV behavioral intervention (≤ 12 mos)	−0.33	0.72*		−0.58	0.56*	
		(0.53 - 0.98)	0.04		(0.35–0.88)	0.01
Perceived moderate- to high-risk of HIV infection	−0.11	0.90		−0.27	0.76	0.30
		(0.65 - 1.24)	0.52		(0.46–1.26)	
Casual, commercial partners (6 mos)	0.22	1.24	0.11	0.50	1.65	0.09
		(0.95 - 1.62)			(0.93–2.90)	
Multiple sexual partnerships (6 mos)	−0.36	0.70*	0.01	−0.21	0.82	0.33
		(0.53 - 0.92)			(0.54–1.23)	
Inconsistent condom use (6 mos)	0.42	1.52*		0.37	1.45	0.07
		(1.14 - 2.02)	0.004		(0.97–2.16)	

Beta: Regression Coefficient; OR = Odds Ration; CI = Confidence Interval; aOR = Adjusted Odds Ratio.

*Statistically significant (*P*-value < 0.05).

^†^Due to colinearity between “receiving free condoms in the last 12 months” and “receiving free water-based lubricant in the last 12 months”, the independent variable “receiving free water-based lubricant in the last 12 months” was removed from the multiple logistic regression models due to lower number of observations. Note: Colinearity does not bias results, but it produces large standard errors in the related independent variables and an effective remedy for Colinearity is to drop an explanatory variable in order to produce a model with significant coefficient estimates for the remaining explanatory variables [14].

Overall, 76.5% of men surveyed had never been voluntarily tested for HIV (never testers). In analyses of aOR, there were statistically significant demographic and behavioural associations with never testing. MSM who reported having a monthly income of less than VND 5 million (equivalent to USD 250) were more likely to be never testers compared to MSM who reported having higher monthly incomes (aOR = 1.99; 95% CI: 1.22–3.23). Study participants who were students were nearly twice as likely to be never testers compared to their non-student counterparts (aOR = 1.88; 95% CI: 1.09–3.24). Men using the Internet more than fifteen hours per week were less likely to be never testers compared to men reporting lower levels of weekly Internet usage (aOR = 0.61; 95% CI: 0.40–0.92). Men who reported ever having participated in a behavioural HIV intervention were less likely to be never testers compared to participants who had participated in such interventions (aOR = 0.56; 95% CI: 0.35–0.88).

There were no statistically significant differences in lifetime HIV testing by age or residential location (i.e. urban centres versus other areas) or MSM archetypes. Engaging in multiple sexual partnerships, with either casual or commercial sex partners in the last six months, as well as being married, were not associated with testing history. In terms of self-perceived HIV risk, being at a medium or higher risk of HIV infection, and self-reported low condom use with any type of sex partner in the last six months were not associated with never having been voluntarily tested for HIV.

## Discussion

There are many benefits of knowing one’s HIV sero-status for both preventing new infections and treatment outcomes [15]. According to Holtgave and McGuire, [16] knowing one’s HIV status and associated counselling are associated with decreased high-risk sexual practices. Marks, Crepaz, Senterfitt, et al. systematically reviewed and analyzed data from the United States, and found that on average, people who knew their HIV-infected status were 53% less likely to have unprotected anal or vaginal sex than people who were unaware of their HIV status [17]. In addition, people who knew their HIV-negative status were 68% less likely to report unprotected penetrative/receptive sex compared to people with unknown status [17]. Beyrer, Wirtz, Walker, et al. assert that “HIV testing provides an excellent opportunity for individual client-focused risk reduction counselling – and for many this session may act as their first such counselling session” [8]. For example, Koblin, Chesney and Coates published findings demonstrating that high-quality regular counselling can significantly reduce risky practices among MSM [18].

There has been scant research specifically focusing on MSM in Viet Nam who have never been tested for HIV, and who represent a particularly important and high-risk population not just in Viet Nam but across the world. In this study, approximately 77% of MSM recruited from approximately a dozen MSM-oriented sexual networking websites in Viet Nam reported never having been voluntarily tested for HIV. Men reporting a monthly income of less than VND 5 million (approximately USD 250) were significantly less likely to have been voluntarily tested. MSM who reported being students were also less likely to have ever been voluntarily tested for HIV. These associations may result from the fact that lower income MSM, many of which are students, may not have access to additional cash to pay for health services such as HIV testing. However, MSM using the Internet more than fifteen hours per week were less likely to be non-testers than those using the Internet less frequently and for shorter periods of time. This finding suggests that MSM social networking sites in Viet Nam may include HIV testing content and/or play an important role in communicating the benefits of HIV testing to MSM-users of these Internet-based platforms.

The association between never being voluntarily tested for HIV and a monthly income of less than VND 5 million is of particular concern. Less affluent MSM may be less likely to have been tested for HIV compared to MSM with higher monthly incomes by virtue of purchasing power and financial opportunities to be tested. Given that men across all income groups reported high percentages of recent unprotected anal intercourse, the higher frequency of never testing among less affluent participants suggests a greater public health response is needed to address the gaps in testing for this population. A significant proportion of MSM in this study reported fear, denial, stigma, and discrimination as barriers to having a voluntary HIV test. It is important to note that 64.8% of MSM surveyed reported knowing where to get tested for HIV, suggesting other potential reasons for alarmingly low HIV testing among study participants. Given that a significant number of MSM cited knowing where to get tested for HIV but reported never having had a voluntary HIV test, further research needs to be conducted on barriers to utilizing existing counselling and testing services in Viet Nam.

The low percentage of voluntary HIV testing found in this sample is cause for concern. HIV testing among this study sample was consistent with data from the 2006 and 2009 rounds of the IBBS in Viet Nam [7,19]. With only 16.6% of men reporting having been voluntarily tested for HIV within the last twelve months, and 50.9% of these actually receiving their test results, the protective benefit of VCT for both an individual and his sexual partner(s) is extremely limited. The 2009 IBBS notes that Ho Chi Minh City (the largest urban centre in Viet Nam and home to the majority of this study’s participants) saw a substantial decrease (from 29% to 19%) among MSM tested and given their HIV results within the last twelve months [7]. As noted in the trend between IBBS rounds, the low levels of testing among MSM in Viet Nam, coupled with a decrease in HIV testing among MSM in Ho Chi Minh City during a period of significant expansion of HIV Testing and Counselling requires further investigation [7].

## Conclusion

We identified critical gaps in HIV testing among a sample of MSM Internet users, particularly among younger MSM living in Viet Nam’s large urban centres. Many men who had never been tested perceived themselves being at low risk for HIV infection; however, their actual risks were much higher as a result of low and inconsistent condom use during anal sex and engaging in multiple and concurrent sexual partnerships. In addition, many participants reported that stigma and discrimination by health professionals was a barrier to being voluntarily tested for HIV. As a result, additional sexual health resources and prevention efforts should be directed toward not only ensuring the provision of stigma/discrimination-free sexual health services for MSM, but also toward increasing awareness about the availability and importance of HIV testing among sexually active MSM. Since a majority of MSM surveyed spent more than seven hours a week on the Internet and were recruited through MSM-specific social networking sites, a key strategy to decrease the number of HIV non-testers would be to engage the owners of MSM social networking sites and work collaboratively to increase the availability of high-quality and engaging HIV testing resources on these sites.

A number of limitations exist when employing an Internet-based self-administered tool to collect data. First, the participants’ responses are uncontrolled, meaning that the respondents may skip questions unintentionally. Second, it may be possible that there will be low site-traffic, meaning that exposure to and response rates of the survey may be affected by a lack of responders. Finally, Internet-based self-administered surveys require that participants have access to the Internet. Individuals who do not have access to the Internet were unable to take part, and therefore individuals without access to the Internet were missed. Although there are as many as 21.5 million Internet users in Viet Nam, [10] many individuals in rural regions may not have the same level of access. Therefore, individuals in urban areas may have been overrepresented in the discussion. Nevertheless, several demographic characteristics from this study were consistent with those from an online study on MSM conducted by Nguyen, Schoenbach, Le, et al. in 2009 [10]. Distribution of age, education level and income were within a 5% range between the two studies, suggesting that this study benefited from a representative sample of Vietnamese MSM Internet users.

## Abbreviations

AIDS: Acquired immunodeficiency syndrome; aOR: Adjusted odds ratio; CI: Confidence interval; FSW: Female sex workers; HIV: Human Immunodeficiency Virus; IBBS: Integrated biological and behavioral study; IBM: International Business Machines Corporation; IDU: Injecting drug users; MOH: Ministry of Health (Viet Nam); MSM: Men who have Sex with Men; MSW: Male sex workers; OR: Odds ratio; SPSS: Statistical product and service solutions; UNAIDS: Joint United Nations Programme on HIV/AIDS; USD: United States Dollar (currency); VCT: Voluntary counseling and testing; VND: Vietnamese Dong (currency).

## Competing interests

The authors declare that they have no competing interests.

## Authors’ contributions

MG developed the study design, protocol, and relevant study tools. MG also implemented the study in 2011, and collected results. MG and QD cleaned, validated and performed statistical analyses. LE validated data output and statistical analyses. SM and PW participated in the design and coordination of this study and helped to draft the manuscript. All authors read and approved the final manuscript.

## Authors’ information

MG has managed and coordinated HIV/AIDS programs funded by the U.S. Government for eight years, specifically working in Botswana, Lesotho, Nigeria and Viet Nam. She currently serves as the U.S. Agency for International Development HIV/AIDS Director in Lesotho. MG is also a Doctorate of Public Health candidate at Flinders University of South Australia. QD has ten years of professional experience in research, monitoring and evaluation for health projects in Vietnam, Ghana, and Lesotho. From 2008 to 2012, she worked on HIV prevention projects targeting most-at-risk populations of HIV, including MSM. She currently serves as the Resident Technical Advisor for the Institute of Health Measurement in Lesotho. LE is an Infectious Disease Epidemiologist currently working with the U.S. Centers for Disease Control and Prevention's Division of Global HIV/AIDS as an ASPH Rosenfield Fellow based in Lesotho, administering technical assistance to PEPFAR Partners and the Ministry of Health to optimize HIV and Tuberculosis surveillance and epidemiology research capacity. Her experience also encompasses the regions of West Africa and the Caribbean - specifically, Guyana and Mali -- where she worked in the areas of HIV and Malaria. LE received a Master of Public Health in Epidemiology from the Tulane University School of Public Health and Tropical Medicine, in New Orleans, Louisiana. SM is a lecturer in the Discipline of Public Health at Flinders University. SM’s research interests include social theories of trust and risk, Theory of Social Quality, and the application of sociological theory to health research. PW is head of the discipline of Public Health at Flinders University. His research interests are socio-spatial inequalities and inequities in health, medicine usage and the provision of health and social care. He also has a particular interest in research around lay and professional perceptions, knowledge and understandings of health, health care, medicines and risk, and how these perceptions vary spatially and by socio-cultural group.

## Pre-publication history

The pre-publication history for this paper can be accessed here:

http://www.biomedcentral.com/1471-2458/13/1236/prepub
